# A community cross-sectional study on oral health status among rural and urban inhabitants of Zambia

**DOI:** 10.1038/s41598-025-24683-4

**Published:** 2025-10-21

**Authors:** Chrispinus Hakimu Mumena, Göran Kjeller, Bengt Hasséus, Daniel Giglio

**Affiliations:** 1 Department of Oncology, Institute of Clinical Sciences, Sahlgrenska Academy, University of Gothenburg, Gothenburg, Sweden; 2 Department of Dental Clinical Sciences, School of Medicine, Copperbelt University, Ndola, Zambia; 3 Department of Oral and Maxillofacial Surgery, Institute of Odontology, Sahlgrenska Academy, University of Gothenburg, Gothenburg, Sweden; 4 Department of Oral Medicine and Pathology, Institute of Odontology, Sahlgrenska Academy, University of Gothenburg, Gothenburg, Sweden; 5 Department of Medicine and Oncology, Southern Älvsborg Hospital, Borås, Sweden

**Keywords:** Oral health status, Caries, Oral potentially malignant disorder, Periodontal disease, Zambia, Dental diseases, Gingivitis, Periodontitis, Dentistry, Epidemiology, Population screening

## Abstract

Information on the prevalence of oral diseases and associated factors in Africa is limited. In this cross-sectional study, we assessed the oral health status in adults living in urban Ndola (*N* = 188) and rural Mansa (*N* = 211) where participants were interviewed on oral health, followed by an oral examination. The chi-square test and logistic regression analysis were used to examine associations between socio-demographic factors, oral disease risk factors, and oral disease presence across urban and rural participants. Dental caries occurred in 72.9% of urban and 47.9% of rural participants (*p* < 0.0001). DMFT was 4.0 in urban participants compared to 2.2 in rural participants (*p* < 0.0001). Gingivitis occurred in 84.5% and 88.1% of urban and rural participants, respectively (*p* = 0.297). Age and habitat were factors significantly associated with dental caries, DMFT, and gingivitis. Smoking and alcohol use were significantly associated with gingivitis, while age, education, smoking, and alcohol use were factors significantly associated with periodontal disease. Only three study participants in the rural population were diagnosed with leukoplakia. In conclusion, dental caries was particularly common in the urban population in Zambia, while gingivitis was high in both the urban and rural populations. Unmet dental care calls for broad interventions to address oral diseases in Zambia.

**Supplementary Information:**

The online version contains supplementary material available at 10.1038/s41598-025-24683-4.

## Introduction

 Oral diseases are a global public health concern affecting 3.9 billion people in the world, with an increase in incidence by almost 21% between 1990 and 2010^1^. Caries of the permanent teeth is the current major public health problem among the non-communicable diseases^2^. Oral health is, however, a neglected problem, especially in African countries, despite that oral diseases share risk factors with other non-communicable diseases such as diabetes and hypertension^2^. Poor oral health is also among the risk factors associated with the development of oral cancer^3^. Other risk factors for oral cancer include tobacco, alcohol, betel quid use, and diet^4^. Oral cancer is in many cases preceded by oral potentially malignant disorders (OPMDs), which include leukoplakia, erythroplakia, oral lichen planus, and submucous fibrosis^5^.

The population in Zambia living below the poverty line is approximately 64.3%^6^. In 2023, Zambia had a GDP per capita of 1331 USD, while Burundi (lowest GDP per capita in the world), Zimbabwe, Botswana (highest GDP per capita on the African continent), and South Africa had a GDP per capita of 193 USD, 2156 USD, 7820 USD, and 6023 USD, respectively^7^. In 2018, there was only one dentist per 100,000 inhabitants working in Zambia^8^. Such a wide gap of dentists to population requires innovative approaches to improve accessibility to oral health services including community oral health outreaches. Despite the gap expressed in dentists to population ratio in most of the African countries, few studies exist on oral health status in the adult population of Zambia, as well as elsewhere in Africa. A systematic review showed a prevalence of dental caries of 36% in the permanent dentition among 12-year-old children in Africa, where Zambia was reported to have the lowest prevalence of 11%^9^. Severine et al. showed that almost half of secondary school adolescents in the Copperbelt province, Zambia, had pre-morbidity stage of dental caries or worse^10^. A population-based study on oral health in Zambia´s eastern neighboring country, Malawi, showed that 49% of people older than 35 had caries, and, the Decayed, Missing, and Filled teeth (DMFT) index in 12, 15, 35–44, 65–74-year-olds was 0.67, 0.71, 3.11, and 6.87, respectively^11^.

Urbanization has an influence on diet and lifestyle and inequalities of oral health care access. These inequalities may be brought by unequal distribution of oral health resources and professionals, where dental services are well concentrated in urban areas compared to rural areas^1^. Currently Ministry of Health statistics shows that about 80% of the Zambians have been affected by oral diseases^12,13^. Understanding the disparity between rural and urban is critical to address and reduce the oral diseases in both rural and urban areas. There is reported improvement of oral health services in rural areas through community outreaches^14^, but yet still there is insufficient population-based studies to inform the current oral health status.

Population-based studies on the prevalence of oral diseases, oral cancer and OPMDs in sub-Saharan African populations are few. The use of tobacco and alcohol abuse are common in sub-Saharan Africa, including Zambia, particularly in uneducated men^15^, constituting significant risk factors for the development of OPMDs and oral cancer^5^. The sale and marketing of tobacco products have also increased in Africa^16^. No studies, to our knowledge, have been conducted on the prevalence of OPMDs in the general or clinic-based populations in Zambia.

The present cross-sectional epidemiological study was conducted to assess oral health in rural and urban Zambia. The prevalence and associated factors of dental caries, gingivitis, periodontal disease, OPMDs, and other oral diseases in the general population were assessed and compared between the rural and urban populations.

## Methods

Ethical approval was obtained from the Tropical Disease Research Centre (TDRC) Institution Review Board (IRB), followed by recommendation from the National Health Research Authority (NHRA). The present cross-sectional study was conducted in the rural area of the Luapula province, in the Mansa district, and in the urban area of the Copperbelt Province, in the Ndola district in Zambia. Mansa district has a population of 205,000 inhabitants whereas 127,000 live in the rural area (for further details on patient selection and recruitment see supplemental methods).

The study population consisted of 399 adults aged 21 years and above, who came to the outreach sites/centers. After obtaining informed consent, 188 participants were recruited from Ndola and 211 participants from Mansa. Participants were interviewed, and a questionnaire was filled out. Confidentiality was assured during the interview process. The registered variables were age, sex, habitat, marital status, education status, dental status, gingival and periodontal status, medical history, tobacco use, and alcohol intake use. The interview was followed by an oral examination. The oral examination was performed by calibrated dentists with the patient sitting in an ordinary office chair, primary school benches and using a flashlight, wooden tongue depressor, dental mirror, and a dental explorer. The oral health form was adapted from the WHO^17^ and structured by improving some of the questions in the language acceptable in the community.

Dental caries was graded as clinically visible and untreated cavities. The DMFT index was used in this study to estimate the caries status (for further details, see supplemental methods)^18^. Gingivitis was scored and graded as absence (0) or presence (1) of bleeding on probing in the gingival sulcus of the index teeth (#16, 11, 26, 36, 31, 46). Conventional tooth pocket probing was not possible due to the meager examination facilities and lack of possibility to inform the study subjects about the procedure. Periodontal disease was defined as a tooth mobility with associated gingival recession and registered and scored grade 1–3, where grade 1 mobility (physiological mobility) indicated tooth mobility of 1 mm in buccolingual direction, grade 2 was tooth mobility of greater than 1 mm without vertical movement of the tooth, and grade 3 was scored when tooth mobility was present in all planes as well as vertically movement^19^. Tooth mobility and gingival recession are robust clinical markers that represent periodontal status when conventional pocket depth measurement is not utilized/applicable, or the focus is on functional implications of periodontal status. This approach is supported by clinical practice and the literature^20–23^. Oral cancer and OPMDs were assessed and distinguished from each other (for further details, see supplemental methods).

### Data management

To assess for statistically significant associations between categories the chi-square test was used. Univariable logistic regression analysis was performed to examine the association of individual variables with specific oral diseases (caries, high vs. low DMFT, gingivitis, and periodontal disease). Subsequently, variables (sex, age, education, cohabitation status, habitat, presence of serious disease, smoking, use of smokeless tobacco, and alcohol use) with a p-value < 0.10 were included in a multivariable logistic regression analysis to evaluate their independent associations with oral diseases. Crude odds ratios (COR) and adjusted odds ratios (AOR) were calculated. Statistical significance was set at a p-value less than 0.05. The mean value ± standard error of the mean (SEM) is given in the text. IBM SPSS Statistics version 26 (IBM Corp., Armonk, NY, USA) and GraphPad Prism program 9.1.0 (GraphPad Software, Inc., San Diego, USA) were used to analyze the data.

## Results

### Sociodemographic data

The sociodemographic data of the cohort has been presented previously^24^. In brief, women constituted 56% and young adults (< 40 years) 59% of the cohort and the average age was 39 ± 0.7 years (*n* = 399). It was more common in rural participants than in urban participants not to have finished primary school (45.5% vs. 28.2%, respectively; *p* < 0.001). Among urban and rural participants, 11.2% and 8.5%, respectively, reported being HIV-positive, while 11.7% of urban participants and 16.6% of rural participants reported not knowing their HIV status (= 0.296). Chronic disease occurred in about one-third of the participants (for details on chronic disease see Supplemental Table 1).

### Oral health status

**Fig. 1:**
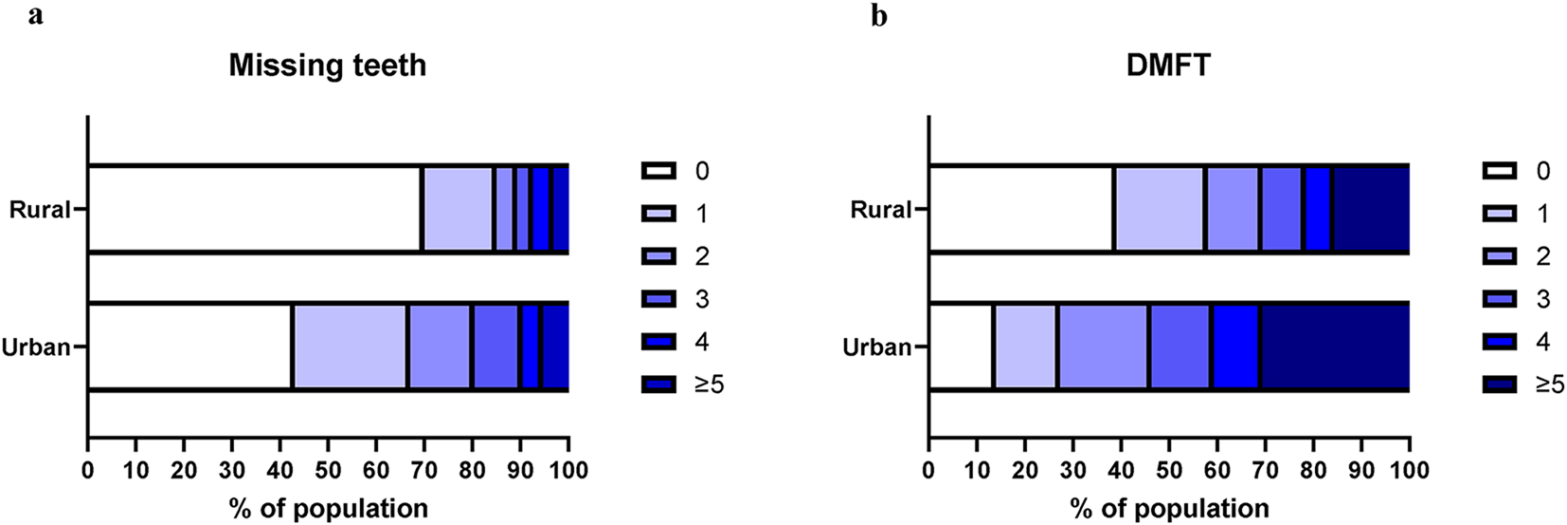
Distribution of rural and urban participants with missing teeth and with different DMFT indexes.

Table 1 Figure 1 and supplemental tables 2-3 show the frequency distribution of oral health status among the rural and urban populations.

**Table 1:** Distribution of the study participants according to oral health status.

Variables	Urban, *N* (%)	Rural, *N* (%)	Significance
Dental Caries Status			< 0.001
No Dental Caries	51 (27.1)	110 (52.1)	
Dental caries	137 (72.9)	101 (47.9)	
**DMFT**			**< 0.001**
Low (< 4.5)	129 (68.6)	175 (82.9)	
High (≥ 4.5)	59 (31.4)	36 (17.1)	
**Periodontal disease**			0.634
No	159 (84.6)	182 (86.3)	
Yes	29 (15.4)	29 (13.8)	
**Gingivitis**			0.297
No	29 (15.4)	25 (11.8)	
Yes	159 (84.5)	186 (88.1)	
**Mucosa lesions**			**0.048**
No	181 (96.3)	206 (97.0)	
Leukoplakia	0 (0.0)	3 (1.4)	
Other lesions	7 (3.7)	2 (0.9)	

Dental caries was more prevalent among urban than rural participants (72.9% and 47.9%, respectively, *p* < 0.001). The DMFT score was significantly higher in urban participants than in rural participants (4.0 and 2.2, respectively; *p* < 0.0001). Likewise, high DMFT (≥ 4.5) was more common among urban participants than rural participants (31.4% and 17.1%, respectively, *p* < 0.001). Only 27.1% of urban participants and 52.1% of rural participants had no decayed teeth, and, among participants, 43.5% had missing teeth. Filled teeth were not encountered in the examined cohort. No participant with missing teeth was found with a dental prosthesis (neither a removable nor a fixed denture was observed).

Gingivitis was common and equally common in urban and rural participants (84.5% and 88.1%, respectively, *p* = 0.297) and all old adults (60 + years) had gingivitis. Urban and rural participants were also equally affected by increased tooth mobility grade 2 and 3, i.e., in 15.4% and 13.8%, respectively, of the participants (*p* = 0.634). Three rural participants (1.4%) had leukoplakia, while seven urban participants (3.7%) and two rural participants (0.9%) had other mucosal lesions such as swelling on the lip, epulis, and oral warts (*p* = 0.048; Table 1).

**Table 2:** Regression analysis of dental caries with sociodemographic variables.

	Univariable analysis		Multivariable analysis OR (95% CI)	
	OR (95% CI)	Significance	OR (95% CI)	Significance
Gender				
Man	Reference			
Woman	0.95 (0.64–1.42)	0.803		
**Age**		**< 0.001**		**< 0.001**
Young adults < 40 years	Reference		Reference	
Middle adults 40–59 years	2.28 (1.42–3.67)	**< 0.001**	2.22 (1.34–3.66)	**0.0019**
Old adults 60 + years	3.03 (1.51–6.51)	**0.0018**	3.11 (1.49–6.46)	**0.0024**
**Education**				
Incomplete primary school or less	Reference			
Completed primary school or more	1.19 (0.79–1.79)	0.414		
**Living with partner**				
Yes	Reference			
No	1.03 (0.64–1.66)	0.899		
**Habitat**				
Urban	Reference		Reference	
Rural	0.34 (0.23–0.52)	**< 0.001**	0.32 (0.21–0.50)	**< 0.001**
**Serious disease**				
No	Reference		Reference	
Yes	0.64 (0.41–1.00.41.00)	0.051	0.75 (0.46–1.23)	0.256
**Smoking**		0.807		
No	Reference			
Yes	1.16 (0.73–1.84)	0.533		
Ex-smoker	1.09 (0.52–2.29)	0.819		
**Smokeless tobacco**				
No	Reference			
Yes	1.29 (0.65–2.55)	0.468		
**Alcohol use**		0.565		
Never/seldom	Reference			
Once a month	1.02 (0.50–2.06)	0.966		
Once a week	1.59 (0.84–3.01)	0.157		
Several times a week	1.38 (0.59–2.08)	0.750		

CI = confidence interval; OR = odds ratio.

Old age (60 + years) was a strong predictor for dental caries [Crude Odds Ratio (COR): 3.03 (1.51–6.51), *p* = 0.0018; Adjusted Odds Ratio (AOR): 3.11 (1.49–6.46), *p* = 0.0024, Table 2], high DMFT [COR: 8.63 (4.33–17.17), *p* < 0.001; AOR: 9.02 (4.39–18.50), *p* < 0.001, Supplemental Table 4], gingivitis (all had gingivitis, Table 3), and periodontal disease [COR: 7.63 (3.54–16.46), *p* < 0.0001; AOR: 5.08 (2.22–11.60), *p* < 0.001, Table 4). To have completed primary school was a protective predictor for gingivitis [COR: 0.25 (0.12–0.55), *p* < 0.001; AOR: 0.29 (0.12–0.66), *p* = 0.0032; Table 3] and periodontal disease [COR: 0.33 (0.19–0.59), *p* < 0.001; AOR: 0.42 (0.22–0.82), *p* = 0.011; Table 4]. Living in rural area was a protective predictor for dental caries [COR: 0.34 (0.23–0.52), *p*< 0.0001; AOR: 0.32 (0.21–0.50), ﻿*p*< 0.0001, Table 2] and for DMFT [COR: 0.45 (0.28–0.72), *p* < 0.0001; AOR: 0.39 (0.23–0.65), *p*< 0.0001, Supplemental Table 4].

**Table 3:** Regression analysis of gingivitis with sociodemographic variables.

	Univariable analysis		Multivariable analysis OR (95% CI)	
	OR (95% CI)	Significance	OR (95% CI)	Significance
Gender				
Man	Reference		Reference	
Woman	0.29 (0.14–0.57)	**< 0.001**	0.46 (0.20–1.04)	0.061
Age		0.0056		0.069
Young adults < 40 years	Reference		Reference	
Middle adults 40–59 years	3.90 (1.70–8.93)	0.0013	2.79 (1.17–6.68)	0.021
Old adults 60 + years	N/A*	N/A*	N/A*	N/A*
Education				
Incomplete primary school or less	Reference		Reference	
Completed primary school or more	0.25 (0.12–0.55)	< 0.001	0.29 (0.12–0.66)	0.0032
Living with partner				
Yes	Reference			
No	0.95 (0.49–1.87)	0.886		
Habitat				
Urban	Reference			
Rural	1.36 (0.76–2.41)	0.298		
Serious disease				
No	Reference		Reference	
Yes	0.36 (0.16–0.78)	0.010	0.60 (0.26–1.42)	0.245
Smoking		< 0.001		0.180
No	Reference		Reference	
Yes	6.10 (2.14–17.35)	< 0.001	2.91 (0.83–10.18)	0.095
Ex-smoker	7.50 (1.00–56.25.00.25)	0.050	3.12 (0.37–26.44)	0.297
Smokeless tobacco				
No	Reference			
Yes	2.04 (0.61–6.87)	0.249		
Alcohol use		0.044		0.408
Never/seldom	Reference		Reference	
Once a month	1.00 (0.39–2.55)	1.000	0.54 (0.18–1.65)	0.278
Once a week	3.20 (0.95–10.74)	0.060	1.37 (0.35–5.31)	0.650
Several times a week	9.40 (1.26–69.94)	0.029	3.24 (0.37–28.07)	0.286

*All participants in the old adult populations were found to have gingivitis. CI = confidence interval; N/A = not applicable (all old adults had gingivitis); OR = odds ratio.

Smoking was a associated with gingivitis and periodontal disease in univariable analysis [COR: 6.10 (2.14–17.35), *p* < 0.001 and COR: 2.47 (1.37–4.49), *p* = 0.0028, respectively] but not in multivariable analysis [AOR: 2.91 (0.83–10.18), *p* = 0.095 and AOR: 1.52 (0.64–3.62), *p* = 0.343, respectively; Tables 3 and 4). Moreover, the use of smokeless tobacco was associated with high DMFT in univariable analysis [COR: 2.36 (1.20–4.66), *p* = 0.013] but not in multivariable analysis [AOR: 1.99 (0.91–4.35), *p* = 0.09, Supplemental Table 4]. Alcohol use several times a week compared to never/seldom alcohol intake was associated with gingivitis in univariable analysis [COR: 9.40 (1.26–69.94), *p* = 0.029] but not in multivariable analysis [AOR: 3.24 (0.37–28.07), *p* = 0.286, Table 3]. Furthermore, alcohol use once a month compared to never/seldom alcohol intake was associated with periodontal disease [COR: 3.71 (1.65–8.34), *p* = 0.0015; AOR: 3.26 (1.29–8.21), *p* = 0.012, Table 4].

**Table 4:** Regression analysis of sociodemographic variables with periodontal disease.

	Univariable analysis		Multivariable analysis OR (95% CI)	
	OR (95% CI)	Significance	OR (95% CI)	Significance
Gender				
Man	Reference			
Woman	0.74 (0.42–1.29)	0.290		
Age		< 0.001		< 0.001
Young adults < 40 years	Reference		Reference	
Middle adults 40–59 years	3.39 (1.71–6.71)	< 0.001	2.89 (1.40–5.95)	0.0041
Old adults 60 + years	7.63 (3.54–16.46)	< 0.001	5.08 (2.22–11.60)	< 0.001
Education				
Incomplete primary school or less	Reference		Reference	
Completed primary school or more	0.33 (0.19–0.59)	< 0.001	0.42 (0.22–0.82)	0.011
Living with partner				
Yes	Reference			
No	1.45 (0.78–2.69)	0.244		
Habitat				
Urban	Reference			
Rural	0.87 (0.50–1.53)	0.634		
Serious disease				
No	Reference		Reference	
Yes	0.44 (0.25–0.77)	0.0043	0.68 (0.35–1.31)	0.249
Smoking		0.012		0.604
No	Reference		Reference	
Yes	2.47 (1.37–4.49)	0.0028	1.52 (0.64–3.62)	0.343
Ex-smoker	1.47 (0.52–4.11)	0.466	1.41 (0.47–4.26)	0.545
Smokeless tobacco				
No	Reference		Reference	
Yes	2.52 (1.18–5.38)	0.017	0.91 (0.33–2.56)	0.865
Alcohol use		0.0056		0.042
Never/seldom	Reference		Reference	
Once a month	3.71 (1.65–8.34)	0.0015	3.26 (1.29–8.21)	0.012
Once a week	1.57 (0.67–3.67)	0.300	1.25 (0.46–3.40)	0.663
Several times a week	2.51 (1.15–5.46)	0.021	2.60 (0.98–6.88)	0.055

CI = confidence interval; OR = odds ratio.

## Discussion

The present study demonstrated a generally poor oral health including untreated dental caries, gingivitis, and periodontal disease in the Zambian population. Gingivitis occurred predominantly among the elderly. Several of the sociodemographic variables such as age, habitat, tobacco use, and alcohol intake were confirmed to predict oral diseases. More than half of the study participants were found with oral diseases which is consistent with previous studies^25^.

This is a unique study for the African continent where information was collected among all adult age groups and among urban and rural inhabitants representative for the Zambian population. Alcohol use was more prevalent among the urban cohort than the rural cohort, which could be related to favorable socioeconomic status in the urban cohort compared to the rural cohort^26^. Speculatively, low income is associated with drinking heavily, while high income is associated with drinking frequency^26^. Tobacco use was similarly common in the rural and urban populations.

The urban cohort was more educated than the rural cohort and education was an independent factor associated with less gingivitis and periodontal disease. Education is associated with better socioeconomic status, and high socioeconomic status is associated with better oral health as demonstrated in previous studies^27^. However, it was observed that dental caries and high DMFT were more common in the urban cohort than the rural cohort in agreement with previous reports^28^. This difference could be due to the impact of urbanization on oral health. Urban environment often leads to higher caries prevalence due to lifestyle and dietary factors such as increased consumption of sugary foods and drinks, changes in oral hygiene practices, and possibly greater access to cariogenic foods. The high prevalence of dental caries among urban participants could also be an example of the effects of nutritional transition to a western diet in African countries, which is linked to an increased prevalence of dental caries^25^.

The prevalence of 47.9% of dental caries in the rural cohort is somewhat higher than the prevalence of 33.9% of dental caries earlier reported in the rural areas of southern Zambia^14^. Since close to half of the Zambian population live in urban areas, the prevalence of untreated caries in the country is estimated to be around 60%. This prevalence is less than among adults in Sudan (87%), similar to the prevalence reported from Eritrea (65%), Malawi (49%) and Rwanda (54–72%) but higher than Tanzania (31%)^1,11,29–32^.

The 2022 to 2026 National Health Strategic Plan estimated that the prevalence of dental caries in Zambia is around 80%, and the findings of this study are in support of this plan showing that existing oral health outreaches are inadequate and confined to selected parts of the country leading to oral health services disparity^33^. Notably, old age and living in a rural area were independent factors significantly associated with dental caries. The urban cohort had a mean DMFT of 4.0, which is twice as much as the rural cohort of 2.2. While decayed and missing teeth were common, no study participants had filled teeth. This suggests long-standing neglect of dental care, with patients often presenting only at advanced stages of disease when restorative treatment is no longer possible. It may also reflect limited awareness of available treatment options for carious teeth and inadequate access to restorative dental care at earlier stages. In most cases in Zambia reporting to the dental clinic for treatment is because of pain when the dental diseases often are advanced^14^.

In the current study, more than three-quarters of both the rural and urban cohorts had gingivitis and all adults above the age of 60 had gingivitis. Gingivitis is an indicator of poor oral hygiene practices^34^. Inaccessibility to oral hygiene devices such as toothbrushes and toothpaste or poor knowledge of the prevention of oral diseases could be factors contributing to poor oral health among the rural participants. Male gender and incompletion of primary school were also factors associated with gingivitis and periodontal disease. The finding that more men than women are affected by gingivitis is consistent with other African studies, including those conducted in South Africa and Tanzania^35,36^. Biological and hormonal factors may here contribute^37^.

While gingivitis was very common, the prevalence of advanced periodontal disease was found to be low. The prevalence was consistent with the prevalence of severe periodontal disease in Zambia reported by WHO and from Ghana but lower than the prevalence of 31.8% of severe periodontitis reported from a general outpatient clinic of a large tertiary hospital in Lagos, Nigeria^38–40^. Age was strongly associated with the presence of periodontal disease. It is important to notice that our cohort constituted predominantly by young individuals (median age 36.5) from a non-hospitalized based cohort, which could have contributed to the low prevalence of periodontal disease compared to aforementioned African studies^38–40^. Participants with a higher education level were also less affected by periodontal disease. Alcohol consumption strongly predicted this condition in the rural and urban cohort. Some reports show that alcohol consumption is associated with periodontal disease^41^, while other studies show no association^42^.

OPMDs were rarely encountered in the studied cohorts. Leukoplakia was demonstrated in the rural population in only three participants, with an overall prevalence of 0.8% in the rural and urban cohorts. This prevalence is lower than the prevalence of 2.4% observed in a cross-sectional study from South Africa^43^.

The strength of this study comes from the fact that information on oral health in the general adult population of Zambia was captured for the first time. Most of the oral health information available is from the studies conducted in children and adolescents. This study has, however, also its limitations. Signs of advanced periodontitis were identified by the presence of gingivitis and increased tooth mobility during screening. Assessment of periodontal pocket depth and obtaining radiographs were not feasible to perform due to the lack of proper examination facilities. Tooth mobility and gingival recession are clinical indicators that may reflect periodontal disease^20,21^. Although examination of tooth pocket probing depth, bleeding from teeth pockets when probing and radiographs are required to set a definite diagnosis of periodontitis^44^, tooth mobility and gingival recessions are strong signs of pathology in the periodontal ligament. Thus, this could lead to an underestimation of the prevalence of periodontitis in the present study.

Furthermore, this study had a slight predominance of women in the rural cohort, which is accounted for by the fact that the study was conducted during working hours, and most of the men were involved in work. The sample size of 399 participants was also relatively small. The selection of participants who were present at the outreach centers may to some extent introduce selection bias by overrepresenting participants who are motivated, have easier access, excluding those who cannot attend.

The findings of this study call for minimal intervention dentistry, focusing on risk assessment, early prevention, and interception of oral diseases to protect and retain natural teeth as long as possible. By improving oral health, it may improve general health and longevity in the population.

In conclusion, oral diseases were common in the Zambian population, particularly among the elderly. The two studied provinces reveal important pattern that are likely representative of the whole country and align with findings reported in the national oral health profiles The oral diseases encountered indicated an unmet need for dental care. Urban habitat, old age, male gender, alcohol abuse, tobacco use, and poor education were confirmed to be associated with the encountered oral health status of the Zambian population.

## Supplementary Information

Below is the link to the electronic supplementary material.

Supplementary Material 1

Supplementary Material 2

## Data Availability

Additional data is available upon request from the Institute of Clinical Sciences at the Sahlgrenska Academy, University of Gothenburg, Medicinaregatan 3 A SE-413 90 Göteborg, Sweden. Email: [klinvet@gu.se](mailto: klinvet@gu.se).
