# Supplementary material for: A community cross-sectional study on oral health status among rural and urban inhabitants of Zambia
Source: Sci Rep. 2025 Oct 21;15:36508. doi: 10.1038/s41598-025-24683-4 (PMC12540755; doi:10.1038/s41598-025-24683-4)
Supplement: Supplementary file 1 — Supplementary Material 1 [file 41598_2025_24683_MOESM1_ESM.docx]

**Supplemental methods**

*Patient selection and recruitment*

The district has 16 wards where the rural wards Chinsuka and Lubende were selected for the study by simple random sampling. Ndola has a population of 375,000 inhabitants. Ndola is divided into 28 wards where simple random sampling was used to choose the urban Dag Hammarskjöld ward. The rural and urban areas were selected by the cluster sampling method (rural *vs.* urban) and the two districts chosen were selected by the stratified sampling method considering low transit activities, socio-economic activities, and social behavior such as alcohol and tobacco use. Data collection involved mobilization of the community through church leaders and priests, while in the residential and work areas announcement was made through public announcement with the use of mega speakers in an open car and on foot house to house in places cars could not access. In each zone, the community members were invited to gather at well-known outreach sites/centers frequently used by health centers in the district. These centers are well known by the community for health-related outreach and research mobilization activities.

*Sample size*

This sample size was obtained by estimating marginal error and precision to 5% and considering that oral health between the two populations is equal with the estimated proportion of the population with oral diseases assumed to be 0.5. Thus, the required sample size was 385 without accounting for non-response. After accounting for non-response of 2%, the adjustment brought the sample size to 393. The inclusion of rural and urban study participants population of 211 and 188 was based on the 2019 National statistics data estimation of 56.9% and 43.1% respectively^1^.

*Clinical assessment*

For DMFT, wisdom teeth were not included, since including them could skew the data and mispresent the actual dental health situation of the population being studied. Dental caries was scored by visually inspection with the aid of a torchlight and a dental explorer was used to confirm clinical caries. DMFT was arbitrary divided into two groups, those below 4 as less severe indicating having 4 decayed/missing or both, and those above 4 as were regarded as having severe decayed/missing or both.

For oral cancer, ulcerations, growth either endophytic or exophytic were evaluated and distinguished from other ulcerations such as aphthous, herpetic, and traumatic ulcers, and acute necrotizing ulcerative gingivitis. Oral candidiasis was distinguished from the leukoplakia considering if the white plaque was easily wiped off using a gauze. Biopsy was performed on any suspicious lesion for histopathological analysis and diagnosis. The research assistants included two oral and maxillofacial surgeons who were trained and calibrated on the diagnosis of the oral lesions using the atlas for oral diseases and conditions in the WHO Basic oral health survey methods[^14^](#_ENREF_14). Examination for other diseases such as dental caries, gingivitis, and periodontitis was performed by three research assistants including two oral and maxillofacial surgeons and one general dentist by using the guidelines in the WHO basic oral health survey methods[^14^](#_ENREF_14).

*References*

1. UNICEF. Country Office Annual Report 2019 Zambia-UNICEF. 2019. <www.unicef.org/media/90446/file/Zambia-2019-COAR.pdf>.
