# Supplementary material for: A community cross-sectional study on oral health status among rural and urban inhabitants of Zambia
Source: Sci Rep. 2025 Oct 21;15:36508. doi: 10.1038/s41598-025-24683-4 (PMC12540755; doi:10.1038/s41598-025-24683-4)
Supplement: Supplementary file 2 — Supplementary Material 2 [file 41598_2025_24683_MOESM2_ESM.doc]

Supplemental table 1. Distribution of chronic diseases among study participants.
	Frequency (n)	Percent (%)	
Cardiovascular disease alone	27	6.8	
Cancer	1	0.3	
Rheumatic disease	3	0.8	
Other serious disease not specified	887	21.8	
Cardiovascular disease + serious disease	3	0.8	
No chronic disease	278	69.5	
Total	399	100.0	

Supplemental table 2. Number of missing teeth among urban and rural participants.
	Missing Teeth	Total	
	0	1	2	3	4	5	6	7	8	9	11	12	16	18	24	26		
Habitat	Urban	N	81	44	25	18	8	1	1	2	2	1	1	1	1	1	0	1	188	
		% 	43.1%	23.4%	13.3%	9.6%	4.3%	0.5%	0.5%	1.1%	1.1%	0.5%	0.5%	0.5%	0.5%	0.5%	0.0%	0.5%	100.0%	
	Rural	N	147	31	9	7	9	0	1	1	1	1	1	2	0	0	1	0	211	
		% 	69.7%	14.7%	4.3%	3.3%	4.3%	0.0%	0.5%	0.5%	0.5%	0.5%	0.5%	0.9%	0.0%	0.0%	0.5%	0.0%	100.0%	
Total	N	228	75	34	25	17	1	2	3	3	2	2	3	1	1	1	1	399	
	% 	57.1%	18.8%	8.5%	6.3%	4.3%	0.3%	0.5%	0.8%	0.8%	0.5%	0.5%	0.8%	0.3%	0.3%	0.3%	0.3%	100.0%	

Supplemental table 3. DMFT score among urban and rural participants.
	DMFT SCORE	Total	
	.00	1.00	2.00	3.00	4.00	5.00	6.00	7.00	8.00	9.00	10.00	11.00	12.00	13.00	14.00	15.00	16.00	19.00	27.00	32.00		
Habitat	Urban	N	26	25	35	24	19	11	6	11	8	4	6	2	5	2	0	1	0	2	1	0	188	
		% 	13.8%	13.3%	18.6%	12.8%	10.1%	5.9%	3.2%	5.9%	4.3%	2.1%	3.2%	1.1%	2.7%	1.1%	0.0%	0.5%	0.0%	1.1%	0.5%	0.0%	100.0%	
	Rural	N	82	39	24	18	12	12	8	3	3	3	0	1	2	1	1	0	1	0	0	1	211	
		% 	38.9%	18.5%	11.4%	8.5%	5.7%	5.7%	3.8%	1.4%	1.4%	1.4%	0.0%	0.5%	0.9%	0.5%	0.5%	0.0%	0.5%	0.0%	0.0%	0.5%	100.0%	
Total	N	108	64	59	42	31	23	14	14	11	7	6	3	7	3	1	1	1	2	1	1	399	
	%	27.1%	16.0%	14.8%	10.5%	7.8%	5.8%	3.5%	3.5%	2.8%	1.8%	1.5%	0.8%	1.8%	0.8%	0.3%	0.3%	0.3%	0.5%	0.3%	0.3%	100.0%	

Supplemental Table 4. Relationship assessed by regression analysis of DMFT ≥4.5 with sociodemographic variables.
	Univariable analysis
	Multivariable analysis
HR (95% CI)	
	OR (95% CI)	Significance	OR (95% CI)	Significance	
Gender			

	
Man	Reference			
Woman	0.92 (0.58-1.46)	0.710		
Age		<0.001		<0.001	
Young adults <40 years	Reference		Reference		
Middle adults 40-59 years	4.76 (2.73-8.29)	<0.001	4.66 (2.64-8.25)	<0.001	
Old adults 60+ years	8.63 (4.33-17.17)	<0.001	9.02 (4.39-18.50)	<0.001	
Education			


	
Incomplete primary school or less	Reference			
Completed primary school or more	1.23 (0.77-1.98)	0.392		
Living with partner				
Yes	Reference			
No	1.24 (0.73-2.11)	0.428		
Habitat				
Urban	Reference		Reference		
Rural	0.45 (0.28-0.72)	<0.001	0.39 (0.23-0.65)	<0.001	
Serious disease			


	
No 	Reference			
Yes	0.77 (0.47-1.25)	0.285		
Smoking		0.156		
No	Reference			
Yes	1.45 (0.87-2.44)	0.157		
Ex-smoker	1.89 (0.86-4.14)	0.112		
Smokeless tobacco				
No	Reference		Reference		
Yes	2.36 (1.20-4.66)	0.013	1.99 (0.91-4.35)	0.09	
Alcohol use		0.760	


	
Never/seldom	Reference			
Once a month	1.16 (0.52-2.60)	0.722		
Once a week	1.19 (0.59-2.38)	0.625		
Several times a week	1.43 (0.72-2.84)	0.306		
CI=confidence interval; OR=odds ratio. 
